# Sperm morphology and performance in relation to postmating prezygotic isolation in two recently diverged passerine species

**DOI:** 10.1038/s41598-022-26101-5

**Published:** 2022-12-24

**Authors:** Manon Poignet, Lucie Baránková, Jiří Reif, Pavel Stopka, Romana Stopková, Michaela Frolikova, Emily R. A. Cramer, Arild Johnsen, Pavel Kverek, Tomasz S. Osiejuk, Katerina Komrskova, Tomáš Albrecht, Radka Reifová

**Affiliations:** 1 Department of Zoology, Faculty of Science, Charles University, Prague, Czech Republic; 2 Institute for Environmental Studies, Faculty of Science, Charles University, Prague, Czech Republic; 3 Department of Zoology, Faculty of Science, Palacký University, Olomouc, Czech Republic; 4 Laboratory of Reproductive Biology, Institute of Biotechnology of the Czech Academy of Sciences, BIOCEV, Vestec, Czech Republic; 5 Natural History Museum, University of Oslo, Oslo, Norway; 6 Vilová 246, 294 02 Kněžmost, Czech Republic; 7 Department of Behavioural Ecology, Institute of Environmental Biology, Faculty of Biology Adam Mickiewicz University, Poznan, Poland; 8 Institute of Vertebrate Biology, Czech Academy of Sciences, Brno, Czech Republic

**Keywords:** Sexual selection, Speciation

## Abstract

Divergence in sperm phenotype and female reproductive environment may be a common source of postmating prezygotic (PMPZ) isolation between species. However, compared to other reproductive barriers it has received much less attention. In this study, we examined sperm morphology and velocity in two hybridizing passerine species, the common nightingale (*Luscinia megarhynchos*) and thrush nightingale (*L. luscinia*). In addition, we for the first time characterized a passerine female reproductive tract fluid proteome. We demonstrate that spermatozoa of the common nightingale have significantly longer and wider midpiece (proximal part of the flagellum containing mitochondria) and longer tail compared to spermatozoa of thrush nightingale. On the other hand, they have significantly shorter and narrower acrosome. Importantly, these differences did not have any effect on sperm velocity. Furthermore, the fluid from the reproductive tract of common nightingale females did not differentially affect velocity of conspecific and heterospecific sperm. Our results indicate that the observed changes in the flagellum and acrosome size are unlikely to contribute to PMPZ isolation through differential sperm velocity of conspecific and heterospecific sperm in the female reproductive tract. However, they could affect other postcopulatory processes, which might be involved in PMPZ isolation, such as sperm storage, longevity or sperm-egg interaction.

## Introduction

Understanding how reproductive barriers originate and accumulate between incipient species is a major goal of evolutionary biology^1,2^. While premating and postzygotic barriers have been studied intensively across a wide range of organisms, postmating prezygotic (PMPZ) isolation has received much less attention, although evidence about its role in speciation is constantly growing^1,3–5^. This is especially true in animals with internal fertilization where the study of postcopulatory sperm behaviour and sperm female interactions is challenging^4,6–8^.

It is commonly assumed that the origin of PMPZ barriers is largely driven by postcopulatory sexual selection, involving sperm competition and cryptic female choice^9–12^. Such selection, often accompanied by sexually antagonistic coevolution, can lead to rapid divergence of male gametes and other components of male ejaculates as well as female reproductive traits between species. Indeed, spermatozoa exhibit an extraordinary diversity in morphology across taxa^13,14^. Also, the genes coding the gamete surface proteins or seminal fluid proteins belong to the fastest evolving genes in the genome^8,15,16^. Despite the growing number of examples showing the importance of PMPZ isolation in speciation^6,15–17^, we still know very little about molecular and physiological mechanisms underlying PMPZ isolation^4^.

Postcopulatory sexual selection can occur at different stages between copulation and fertilization^3,18,19^. One of the most important determinants of fertilization success, both under competitive and non-competitive scenarios, is sperm velocity^10,20–22^. Sperm velocity often correlates positively with sperm length^23–26^, which is mostly determined by length of the flagellum. It is generally assumed that a longer flagellum promotes sperm propulsion^24,27–29^. At the same time midpiece length and width, a part of the flagellum containing mitochondria, is assumed to determine the amount of energy generated through oxidative phosphorylation necessary for sperm movement^24,30,31^. On the other hand, the size of the sperm head can affect sperm velocity negatively due to the increased level of drag in the viscous environment of the female reproductive tract^24,32,33^. The relationship between the total sperm length and velocity is thus not straightforward and there are examples where shorter spermatozoa are faster than the longer ones^34–36^.

The protein composition of the female reproductive tract fluids, which form an environment for sperm on their way to the egg, may further modulate sperm velocity and the likelihood of the egg fertilization^37^. Given the intimate co-evolution between sperm traits and female reproductive environment, conspecific sperm may perform better than the heterospecific sperm in the female reproductive tract^38,39^. However, mechanisms of how heterospecific and conspecific fluids affect sperm behaviour are not sufficiently understood^6,34,40–43^. Moreover, protein composition of the female reproductive tract fluids has been characterized only in a handful of species^4,44,45^ and in many organisms, including passerine birds, the proteome of the female reproductive tract fluid is still unknown.

Sperm velocity is, however, not the only determinant of sperm competitiveness. In many species, including birds, sperm storage in the female reproductive tract significantly affects sperm fertilization success^46–49^. Given that size and shape of the sperm storage organs often closely co-evolve with sperm size^46,50–52^, differential sperm storage of conspecific and heterospecific sperm may contribute to PMPZ isolation. In addition, the ability of sperm to bind to and penetrate the glycoprotein perivitelline layer and fuse with the plasma membrane of the egg is crucial for the whole fertilization process^53^. The acrosome, an apical vesicle that evolves in the anterior part of the sperm head, contains enzymes necessary for the sperm to penetrate the inner perivitelline layer^54^ during the process called the acrosome reaction^55^. The difference in size and shape of the acrosome among species may thus also contribute to PMPZ isolation.

**Figure 1:**
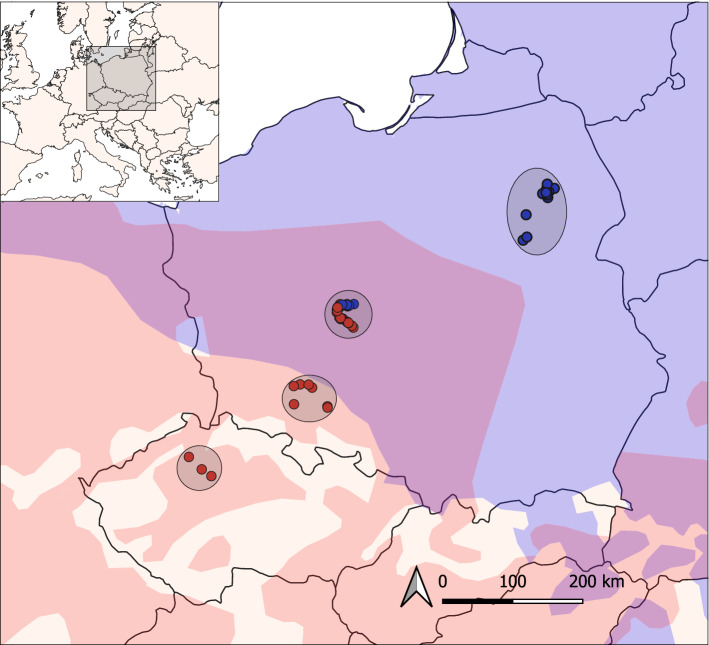
Sampling localities of common nightingales (red) and thrush nightingales (blue) in Poland and the Czech Republic. The distribution areas of common and thrush nightingale are in red and blue, respectively. The sympatric area, where both species co-occur, is in violet. The map was generated using QGIS v.3.10 (http://qgis.osgeo.org) with species ranges distribution from BirdLife International^67,68^ and modified following Reif et al.^60^.

In the present study, we investigated sperm morphology and performance in the context of PMPZ isolation in two closely related passerine species, the common nightingale (*Luscinia megarhynchos*, Brehm 1831) and the thrush nightingale (*Luscinia luscinia*, Linnaeus 1758). Passerine sperm are unusual in their corkscrew shape and very long midpiece forming most of the flagellum length^13,14,24^. The two nightingale species diverged approximately 1.8 Mya^56^ and currently occasionally hybridize in a secondary contact zone spanning across Central and Eastern Europe^57–59^ (Fig. 1). The species are partially separated ecologically^60–62^ and female-limited hybrid sterility contributes to postzygotic isolation^63,64^. Both nightingale species are socially monogamous but sexually promiscuous, with a higher level of extra-pair paternity in the common nightingale (~ 24%) than in the thrush nightingale (~ 6%)^65^. Albrecht et al.^66^ found marked divergence in sperm length between the species, mostly driven by longer midpiece in the common nightingale compared to thrush nightingale, which could potentially contribute to PMPZ isolation between the species. The species also slightly differed in the sperm head length^66^. Interestingly the divergence in the sperm head length was higher in sympatry than in allopatry, indicating that reinforcement at the postcopulatory level might have occurred in the two species. However, that study did not distinguish the size of the acrosome and the nucleus, the two compartments of the sperm head, so it is unclear whether only one or both of those compartments drove the head length divergence. It is also unclear whether the differences in sperm morphology affect the sperm velocity.

Here we studied the potential effect of sperm divergence on PMPZ isolation between the nightingale species by evaluating the relationship between sperm morphology and its performance in an environment of the conspecific and heterospecific female reproductive tract. Specifically, we (1) performed a detailed analysis of sperm morphology, separately measuring nucleus and acrosome length, in both species using confocal and scanning electron microscopy and (2) compared the sperm velocity between species in a cell culture medium. In addition, we (3) characterized the proteomic composition of fluid from the distal part of the female reproductive tract and (4) tested whether it affects behaviour of conspecific and heterospecific sperm.

## Results

### Differences in sperm morphology between species

**Figure 2:**
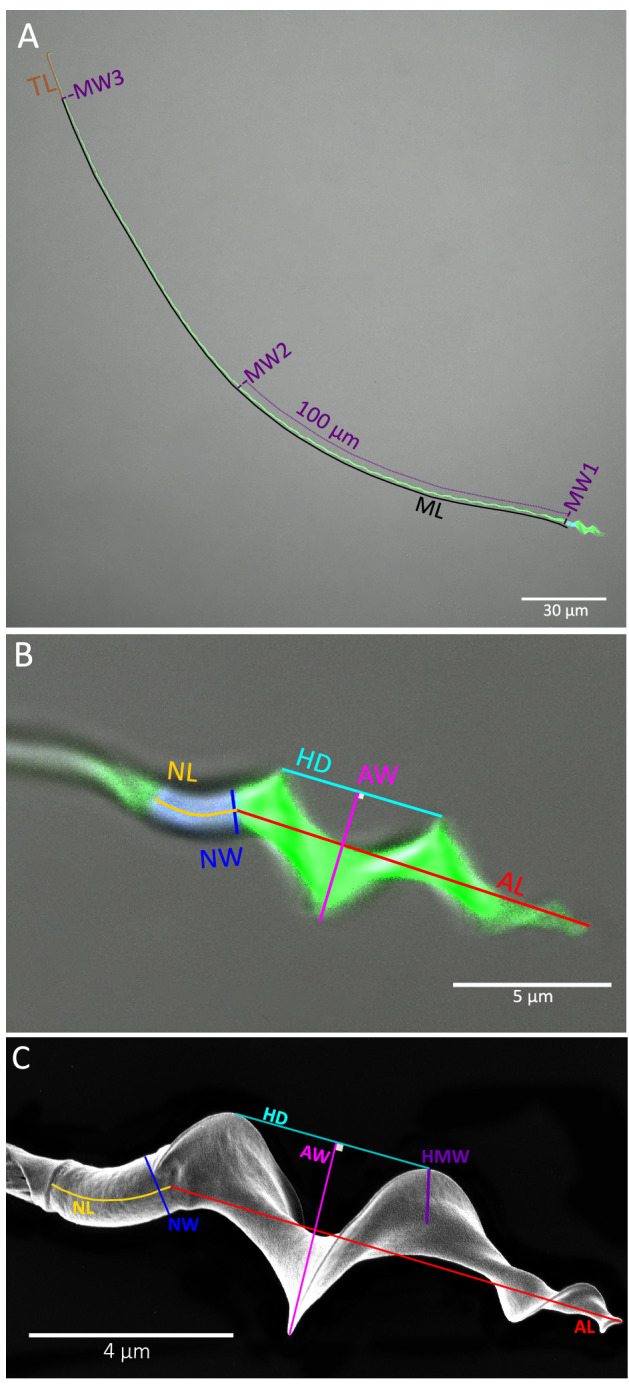
Sperm morphological traits measured on photos from confocal (**A**, **B**) and scanning electron microscope (**C**). On photos from confocal microscope, the flagellum and the acrosome are labelled using MitoTracker (green) and the nucleus by DAPI (blue). The following traits were measured on the flagellum: midpiece length (ML), midpiece width (MW1, MW2, MW3), and tail length (TL). On the sperm head we measured: acrosome length (AL), acrosome width (AW), nucleus length (NL), nucleus width (NW), helix distance (HD), and helical membrane width (HMW). Displayed images are of representative common nightingale sperm with the background modified to remove debris.

Sperm morphology in the common nightingale (hereafter CN) and the thrush nightingale (hereafter TN) was studied using (1) confocal microscopy of the immunostained spermatozoa, which allowed us to distinguish different compartments of the sperm head and flagellum, and (2) scanning electron microscopy, which provides more details about the surface and shape of the sperm head. Sperm morphology was analysed in ten sympatric and ten allopatric individuals per species in the case of confocal microscopy, and in five sympatric individuals per species in the case of scanning electron microscopy. Ten sperm were analysed per individual. The following sperm traits were measured on the flagellum: midpiece length (ML), midpiece width (1) at the proximal tip (MW1), (2) 100 µm from the proximal tip (MW2), (3) at the distal tip (MW3), and tail length (TL) (Fig. 2A). On the sperm head we measured: acrosome length (AL), acrosome width (AW), helix distance (HD), nucleus length (NL) and nucleus width (NW) (Fig. 2B). On images from the scanning electron microscopy, we additionally measured the helical membrane width (HMW) on the acrosome (Fig. 2C). The estimates of the within-sperm measurement repeatability were higher than 0.63 for ML, MW3, TL, AL, AW and in all cases significant (Supplementary Table S1). For MW1, MW2, HD, NL and NW the repeatability estimates were slightly lower, partly because the measured dimensions of these traits are generally smaller, and partly because the helical structure of the sperm does not provide clear landmarks for measuring some of these variables. The measurement repeatability was, however, always higher than 0.30 and in all cases significant (Supplementary Table S1). The correlation coefficients among sperm traits were small for both confocal (CN: from − 0.31 to 0.49; TN: from − 0.37 to 0.28) (Supplementary Fig. S1) and scanning electron (CN: from − 0.34 to 0.56; TN: from − 0.54 to 0.38) microscopy (Supplementary Fig. S2). Therefore, all sperm traits were analysed independently except for MW1, MW2 and MW3 for which we took the average value (MW) providing the average width of whole midpiece of the sperm tail.

**Figure 3:**
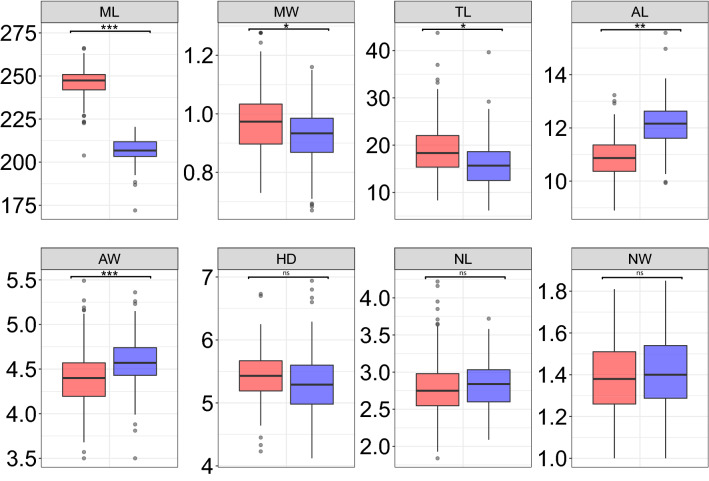
Differences in the sperm traits between the common nightingale (red) and thrush nightingale (blue) using confocal microscopy data. Following traits were compared: midpiece length (ML), midpiece width (MW), tail length (TL), acrosome length (AL), acrosome width (AW), helix distance (HD), nucleus length (NL) and nucleus width (NW). All measurements are shown in µm. Medians, quartiles, 1.5 quartile range and outliers (based on 200 measured sperm per each species) are shown. P-value: ns = P > 0.05; * = P ≤ 0.05; ** = P ≤ 0.01; *** = P ≤ 0.001.

Linear mixed models with individual sperm traits as response variables and species, region and their interaction as explanatory variables revealed no significant effect of region or species and region interaction on any of the measured sperm traits (Supplementary Table S2). We thus simplified the models by excluding the region and tested just for the effect of species on individual sperm traits. Analyses of the sperm morphology data from the confocal microscope confirmed that the two nightingale species differ markedly in the flagellum length and that this difference is mostly driven by changes in the midpiece length^66^. The CN had a much longer midpiece (mean ± sd = 246.15 ± 8.34 µm) compared to TN (mean ± sd = 206.93 ± 6.87 µm) (Fig. 3, Table 1, Supplementary Table S3A). The midpiece of the CN was also slightly, but significantly, wider compared to TN (Fig. 3, Table 1). The length of the tail was slightly but significantly longer in the CN compared to the TN (Fig. 3, Table 1).

**Table 1:** Reduced linear mixed models (with excluded effect of region) testing for the effect of species on sperm morphological traits.

Model terms	Estimate ± SE	F	P	P-adjusted
**(i) Flagellum traits**				
Midpiece length				
Intercept	246.27 ± 1.19	–	–	–
Species	− 39.27 ± 1.68	546.85	** < 0.001**	** < 0.001**
Midpiece width				
Intercept	0.99 ± 0.02	–	–	–
Species	− 0.06 ± 0.02	5.96	**0.02**	**0.032**
Tail length				
Intercept	18.96 ± 0.88	–	–	–
Species	− 2.99 ± 1.23	5.89	**0.02**	**0.032**
**(ii) Head traits**				
Acrosome length				
Intercept	10.89 ± 0.11	–	–	–
Species	1.27 ± 0.16	66.27	** < 0.001**	** < 0.001**
Acrosome width				
Intercept	4.39 ± 0.03	–	–	–
Species	0.18 ± 0.05	13.35	** < 0.001**	**0.002**
Helix distance				
Intercept	5.43 ± 0.05	–	–	–
Species	− 0.13 ± 0.08	2.95	0.10	0.13
Nucleus length				
Intercept	2.79 ± 0.06	−	−	−
Species	0.05 ± 0.08	0.34	0.56	0.56
Nucleus width				
Intercept	1.39 ± 0.02	–	–	–
Species	0.02 ± 0.02	0.96	0.33	0.38

The common nightingale was a reference species. Measurements were made on photos from confocal microscope. Estimates, standard errors (± SE), F-stats, P-values and adjusted P-value based on Benjamini–Hochberg correction (P-adjusted) are shown for all models. Significant P-values are in bold.

Regarding the head size measurements, we found significant differences in the acrosome size between species, with a longer and wider acrosome in the TN compared to the CN (Fig. 3, Table 1, Supplementary Table S3A). This difference was confirmed by data from the scanning electron microscope (Supplementary Tables S3B, S4), although in the case of acrosome width, the difference between species was not significant, possibly due to the lower sample size available for scanning electron microscopy. The length and width of the nucleus showed no significant differences between the species neither using data from confocal (Fig. 3, Table 1) nor scanning electron microscope (Supplementary Table S4). The helix membrane width and helix distance were also not significantly different between the two species (Table 1, Supplementary Table S4).

### Sperm swimming performance in the DMEM cell culture medium

To evaluate the impact of sperm morphology differences on sperm swimming performance, we measured sperm velocity, estimated as curvilinear velocity (VCL), in the cell culture medium (Dulbecco’s Modified Eagle’s Medium (DMEM)). Sperm velocity was evaluated in 19 CN and 15 TN males. Despite the marked differences in sperm morphology, we found no significant difference in the sperm velocity between the species (P = 0.94, Table 2). The sperm velocity was, however, affected by the number of motile sperm in the sample (P = 0.02, Table 2). We thus tested whether the number of motile sperm differed between the species, but no significant difference was observed (P = 0.54, Table 2).

**Table 2:** Linear mixed models testing for the effect of (i) species identity and total number of motile sperm on sperm velocity in the cell culture medium (DMEM), and (ii) species identity on the number of motile sperm.

Model terms	Estimate ± SE	F	P
**(i) VCL**			
Intercept	59.64 ± 15.88	–	–
Species	1.96 ± 5.73	0.006	0.94
Number of motile sperm	7.59 ± 3.13	5.88	**0.02**
**(ii) Number of motile sperm**			
Intercept	4.93 ± 0.21	–	–
Species	-0.20 ± 0.32	0.39	0.54

In both models, the common nightingale was set as the reference species. The estimates, standard errors (± SE), F-stats and P-values are shown, with significant P-values in bold.

### Proteomic composition of the female reproductive tract fluid

**Figure 4:**
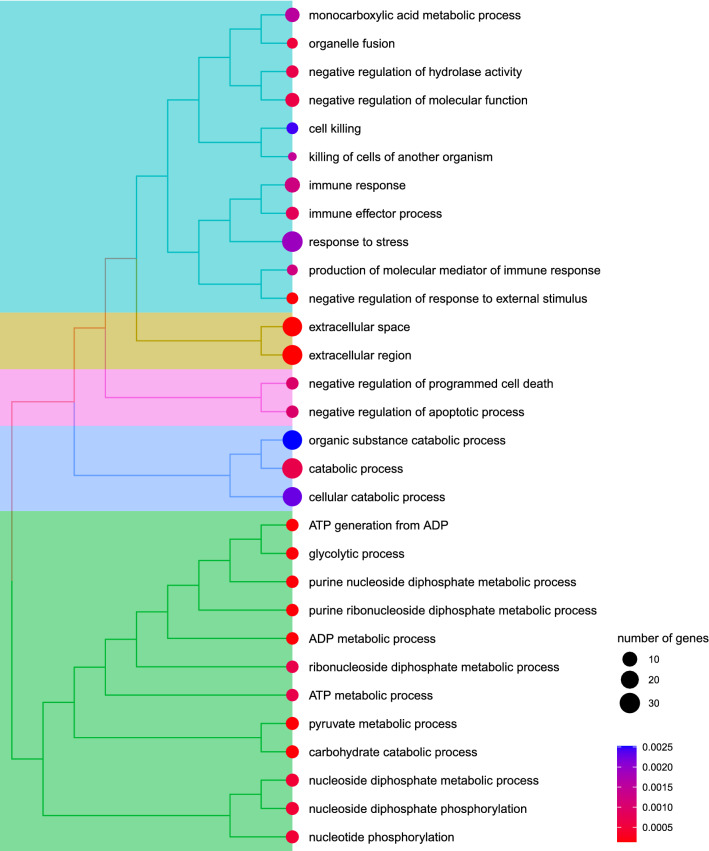
Gene ontology (GO) enrichment analysis of the common nightingale female reproductive tract fluid proteomes. Gene ontology searches revealed that highly expressed proteins are extracellular soluble proteins mostly involved in the maintenance of tissue homeostasis, antimicrobial defence and ATP metabolism. Numbers of enriched genes are reflected by the circle size while Benjamini–Hochberg corrected p-values are scaled from red to blue. Hierarchical clustering of enriched terms relies on the pairwise similarities of the enriched terms.

Fluid samples from the distal part of the female reproductive tract (i.e., the part of the tract that is in the first contact with sperm and corresponds to cloaca and distal part of vagina) coming from four CN females were used in this analysis. A total of 563 different proteins with at least two unique peptides were identified in this female fluid (Supplementary Table S5). To deconstruct the physiological role of this fluid, we searched for significantly enriched GO terms among the highly abundant proteins across all individuals as defined by the mixture models (posterior P-value < 0.1). These highly expressed proteins include for example: KRT19, KRT75L2, ACTB, ALB, PRSS3, AKR1B10, KRT75, LTF, KRT10, UBB, LYZ, ACTC1, HSPA8, KRT4, GAPDH, NMRAL1 and MIF. In the cellular component category, the extracellular space/region was among the significantly enriched GO terms (P < 0.002, Fig. 4). This is reassuring as this is typical for the soluble fraction of secretions. Within the significantly enriched GO terms related to biological and molecular processes, there was a striking prevalence of terms related to immune response, cell killing and response to stress (Fig. 4), which suggests that the primary role of female fluid in the distal part of the reproductive tract is mostly immuno-protective, providing females stable homeostasis of the reproductive organ during breeding. However, there were also significant GO terms related to ADP/ATP metabolism and glycolysis, cellular catabolic process and negative regulation of apoptosis suggesting that there might be other, as of yet unspecified, functions of female fluids, which could, besides other things, affect sperm performance in the female reproductive tract.

Among all identified proteins, regardless of their abundance, there was a group of moderately expressed proteins which belongs to a protein family called Calycins; namely the fatty-acid binding proteins FABP3-7, and apolipoproteins (APOA1, APOA4). All these proteins play important protective roles in mucosal tissues due to their capacity to bind and transport lipids and other lipophilic molecules (including radical oxygen species) across many vertebrate taxa^69^. Similarly, we detected 13 proteasomal proteins (PSMA1-7, PSMB1, 2, 4, 6, PSMC2, and PSMD11), which provide further support for high metabolic/catabolic activity in female reproductive organs. We detected seven members of the annexin family (ANXA1, 2, 4, 5, 6, 7, and 11) which is known to inhibit inflammation^45^. Additionally, most of the highly expressed and many of the moderately expressed proteins limit bacterial growth and maintain the stable tissue homeostasis, which might, among other things, influence sperm survival and successful fertilization.

### The impact of female reproductive tract fluid on velocity of conspecific and heterospecific spermatozoa

**Figure 5:**
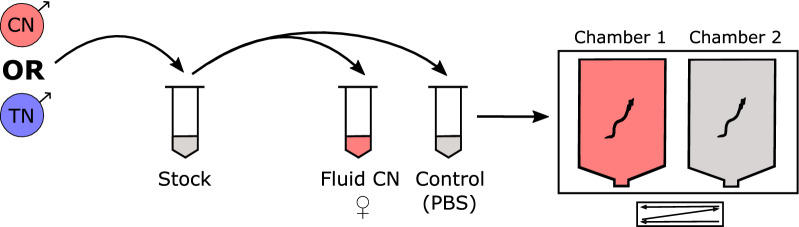
Design of the experiment comparing velocity of conspecific and heterospecific sperm in the fluid from the female reproductive tract. Velocity of conspecific and heterospecific sperm was measured in the common nightingale (CN) female fluid and directly compared with the velocity in the control environment (physiological saline buffer, PBS) using one microscopic slide with two chambers. TN, thrush nightingale; CN, common nightingale.

We further tested whether the fluid from the distal part of the female reproductive tract affects the sperm swimming performance and whether spermatozoa swim slower in the female fluid from heterospecific than conspecific females, which would indicate the presence of PMPZ isolation between the species. To do so CN and TN sperm were recorded, separately for each species, in the physiological saline buffer (PBS) with the addition of the female fluid. To provide a comparison point, sperm from each male was also simultaneously recorded in a pure PBS. The experimental design is shown in Fig. 5. In each experiment, we used a female fluid of one CN female together with a sperm sample from one CN male and one TN male (Fig. 5). We conducted 13 such experiments. To avoid pseudoreplication, each fluid and sperm sample was used only in one experiment.

We found that sperm velocity was significantly affected by the presence of the female fluid in the PBS (P = 0.02, Table 3). Spermatozoa had lower velocity in PBS with the addition of the female fluid than in pure PBS (Supplementary Fig. S3). However, the presence of the female fluid affected the velocity of conspecific and heterospecific spermatozoa in the same way (there was no significant interaction between the species from which the sperm originated and the environment) (P = 0.84, Table 3, Supplementary Fig. S3). The number of motile spermatozoa in the sample did not have a significant effect on sperm velocity in this experiment (Table 3).

**Table 3:** The linear mixed model testing for the effect of the species identity, environment (female fluid vs. PBS) and their interaction on sperm velocity (VCL).

Model terms	Estimate ± SE	F	P
**VCL**			
Intercept	60.68 ± 6.96	–	–
Species	− 4.52 ± 4.73	1.90	0.20
Environment	7.53 ± 4.21	5.62	**0.02**
Number of motile sperm	1.47 ± 1.57	0.87	0.35
Start time	− 0.06 ± 0.04	1.76	0.19
Species × environment	− 1.19 ± 5.80	0.04	0.84

The number of motile sperm and starting time of the particular recording in sec were included in the model as covariates. The common nightingale species and female fluid environment were set as a reference. The estimates, standard errors (± SE), F-stats and P-values are shown, with significant P-values in bold.

It was unfortunately not possible to capture TN females during the early breeding season in large enough numbers to enable us to examine sperm performance in TN female fluid.

## Discussion

It is commonly assumed that rapid divergence in sperm traits between species could play an important role in PMPZ isolation between species^70^. However, empirical evidence for PMPZ isolation is still relatively scarce and specific mechanisms behind PMPZ isolation are largely unexplored, especially in vertebrates^6,71^. In this study, we performed a detailed analysis of sperm morphology in two closely related and hybridizing passerine species, the common and thrush nightingale, which differ in levels of postcopulatory sexual selection^65^, and evaluated the relationship between sperm morphology and velocity. In addition, we analysed the protein composition of fluid from the distal part of the female reproductive tract and evaluated its impact on the velocity of conspecific and heterospecific sperm.

In agreement with the previous study^66^, we found that the two nightingale species exhibit a marked divergence in the length of the midpiece—the largest part of the flagellum. The common nightingale, which appears to be the more promiscuous species^65^, had a longer midpiece (~ 246 µm) than the thrush nightingale (~ 207 µm). Such divergence is striking given only 1.8 million years of divergence of these species. High levels of divergence in the midpiece length were also observed in *Luscinia svecica* subspecies^72^, suggesting a particularly rapid evolution of sperm morphology in this genus. The common nightingale had, at the same time, slightly wider midpiece than the thrush nightingale, suggesting that the common nightingale had an overall higher midpiece volume. This is consistent with recent study across multiple passerine species showing that sperm with longer midpiece tend to have also wider midpiece^73^. The tail length was also slightly longer in the common nightingale making the total flagellum length longer in this species. Given that the main function of the midpiece is the production of ATP through oxidative phosphorylation in mitochondria and longer flagellum can provide stronger forward thrust, it is tempting to speculate that longer flagellum could increase velocity of the common nightingale’s sperm. Indeed, it has been shown that sperm length (which is strongly correlated with midpiece length in passerine birds) tends to be positively associated with extra-pair paternity levels in passerines^12,74,75^ and that longer sperm usually have higher velocity, although this relationship appears not to be universal^35,75,76^. Here, however, we found no difference in the sperm velocity between the common and thrush nightingale sperm. It is thus possible that the striking difference in the midpiece length (and total flagellum length) between the species is driven by other forces. For example, common nightingale sperm with larger midpieces and tail may have higher longevity in the female reproductive tract than thrush nightingale sperm. Or co-evolution between the sperm length and the size of the sperm storage tubules in females, which have been observed across the passerine birds^46^, may drive changes in sperm morphology.

Besides divergence in the midpiece and tail length, we found that these two species differ in the acrosome size with the sperm of thrush nightingale having significantly longer and wider acrosomes compared to the common nightingale. This suggests that previously observed difference in the sperm head length between the nightingale species^66^ is caused by an increase of the acrosome size in the thrush nightingale. The previous study based on extensive sampling of both species across the hybrid zone (112 samples) showed a higher divergence in the sperm head length in sympatry compared to allopatry^66^. Here, we have not found a significant difference in the acrosome or nucleus size between regions, which may be, however, caused by much low sample size (40 samples). Sperm heads with larger acrosome are theoretically expected to reduce the sperm velocity, as bigger sperm head can yield greater drag forces^24,32,33^. However, Støstad et al.^14^, who measured passerine-specific sperm head parameters in a greater detail than previous studies, found that longer acrosomes with wider helical membranes correlated with faster swimming speed across species. It is thus possible that the longer midpiece in the common nightingale and the larger acrosome in the thrush nightingale have a similar positive effect on sperm velocity resulting in the same sperm swimming speed in both species.

Nevertheless, the primary role of the acrosome is to mediate the fusion of the sperm with the egg. The acrosome contains specific molecules necessary for sperm-egg binding as well as lytic enzymes needed for the penetration of the sperm through the inner perivitelline layer to enable the gametes’ membrane fusion^54,77^. The divergence in the acrosome size among species might thus indicate differences in the interaction between the sperm and the egg’s glycoprotein surroundings prior to the fertilization. It is for example possible that the nightingale species differ in thickness of the glycoprotein layer around the eggs and differences in acrosome sizes between the species reflect the ease with which sperm can penetrate it^78^. Further research is needed to test these possibilities.

The environment of the female reproductive tract can significantly affect sperm performance and the probability to reach the egg^79^. In birds, the distal part of the reproductive tract appears to be the crucial site for sperm selection^80^ as only about 1–2% of the spermatozoa successfully move through the cloaca and vagina and enter the sperm-storage tubules located at the uterovaginal junction^81,82^. Our study for the first time addressed the protein composition of the fluid from the distal part of female reproductive tract, including the cloaca and distal part of vagina, in passerine birds. We identified 563 different proteins in the fluid from the distal part of the female reproductive tract. Analysis of their function indicated, as expected, that the main function of the female fluid proteins lies in the defence against microorganisms and providing protective environment for the female. It is, however, possible that some functions may also affect the performance of male gametes. The enrichment of ATP generation from ADP, glycolysis and cellular catabolic process, for example, suggest that the fluid may also influence the sperm motility. In our experiment, we found that the sperm velocity was significantly reduced by the presence of the female fluid in the PBS. Although it is hard to presume the mechanism behind, we can imagine that the protective effect of the female fluids against harmful microorganisms can as a side effect select against sperm. It can be, however, also just an effect of increased viscosity or some other characteristic of the liquid environment changed by the addition of the female fluid^76^.

Importantly, there was, however, no difference in how the common nightingale female fluid affected the velocity of conspecific and heterospecific spermatozoa, which suggests that the fluid from the distal part of the female reproductive tract does not discriminate conspecific and heterospecific sperm in nightingales. These results are consistent with similar experiments in four other pairs of passerine species with more or less divergent sperm phenotypes: house sparrows (*Passer domesticus*) vs. Spanish sparrows (*P. hispaniolensis*), barn swallows *Hirundo rustica* versus sand martins *Riparia riparia*, two subspecies of bluethroats, *Luscinia svecica svecica* versus *L. s. namnetum*, and great tits *Parus major* versus blue tits *Cyanistes caeruleus*, in which sperm performed equally in conspecific and heterospecific female fluids^42,43^. By contrast, in two closely related flycatcher species, *Ficedula hypoleuca* and *F. albicollis*, which do not show clear differences in sperm morphology, the sperm velocity was significantly reduced in heterospecific female fluid compared to conspecific fluid^6^. This effect was, however, seen only in one direction of the cross, with reduced sperm performance being possibly linked to prior exposure to heterospecific sperm by females who are most likely to have been constrained to mate with heterospecifics^6^. Here, we were only able to test the effects of common nightingale female’s fluid on sperm, so we cannot rule out that thrush nightingale female fluid could have a differential effect on conspecific and heterospecific sperm. Moreover, since the conditions in the female reproductive tract are more complex than in our *in-vitro* experiments, it is possible that some factors not reflected in our experiments may affect velocities of conspecific and heterospecific sperm under natural conditions. Finally, the two nightingale species may differ in total sperm counts in ejaculates, which could also influence the success of fertilization and levels of PMPZ isolation between the species. Unfortunately, the method of obtaining ejaculate samples, which was used in this study, is not suitable for inferring ejaculate sizes or sperm numbers and we thus could not address this question.

## Conclusions

We found substantial differences in sperm morphology between two recently diverged nightingale species. This divergence in sperm morphology, however, did not have any effect on sperm velocity in a cell culture medium. Although the proteome analysis of the female reproductive tract fluid revealed the potential of the female fluid to affect the sperm motility, we found no difference in the velocity of conspecific and heterospecific sperm in the fluid. Our results indicate that the observed divergence in sperm morphology is unlikely to contribute to PMPZ isolation between the species via differential speed of conspecific and heterospecific sperm in the female reproductive tract. Nevertheless, it is possible that the differences in sperm morphology could affect the likelihood of storage in the sperm storage tubules or the ability of the sperm to penetrate the inner perivitelline layer and fertilize the egg. Further studies will be needed to understand the potential mechanisms of PMPZ isolation in nightingales and generally in birds.

## Material and methods

### Sampling

The sampling of CN and TN individuals was conducted at the beginning of their breeding season (the first half of May) between 2014 and 2019. The female individuals were captured in the period between the 6th and 15th of May, and only females in the early nesting phase (verified by the development of brood patches) when copulation occurs^83^ were included in the experiment. Birds were captured in allopatric regions (South-Western Poland and the Czech Republic for the CN and North-Eastern Poland for TN) as well as sympatric region (central Poland) (Fig. 1). Individuals were captured using mist nets or collapsible traps accompanied by the playback of conspecific song. Each individual was ringed, measured, and sexed, and species was determined using species-specific morphological characteristics^84,85^. In total, 65 and 48 individuals of CN and TN were captured, respectively (Supplementary Table S6).

From male individuals, sperm ejaculates were obtained by gentle massage of the cloaca^86^ and collected using glass capillaries preheated to 40 °C. Sperm samples were either directly used in a sperm velocity experiment (as detailed below) or mixed with about 20 µl PBS and stored at 4 °C in 10% formalin or paraformaldehyde for later sperm morphology analysis. From female individuals, fluid from the distal part of the female reproductive tract was collected as follows^42^. The exterior surface of the female cloaca was swabbed with a cotton swab soaked in 96% ethanol and allowed to air dry. The cloaca was then gently massaged to expose the mucosal surface and a small volume (5 µl) of sterile PBS was pipetted in. After 10 s, PBS from the cloaca was collected by pipette and dropped into a cryotube. This process was done three times to obtain a total of 15 µl of female fluid for each individual. The 15 µl of female fluid was mixed in the cryotube and then divided into three cryotubes of 5 µl each and frozen in liquid nitrogen for later proteomic analysis and sperm velocity experiments. 5 µl of pure PBS was also frozen to obtain the same initial condition for both female fluid and PBS (control) sperm velocity experiments. All fieldwork procedures were approved by the Local Ethic Committee for Scientific Experiments on Animals in Poznan, Poland (permissions no. 27/2008 36/2010 and 17/2015) and by the Ethic Committee of the Faculty of Science, Charles University (permission no. 9833/2007–30). All methods were carried out in accordance with relevant regulations and guidelines and reported in accordance with ARRIVE guidelines (https://arriveguidelines.org).

### Analysis of sperm morphology

#### Confocal microscopy

Sperm samples stored in formalin were centrifuged (25,000 rpm, 5 min at 25 °C) and 20 µl of pellet was transferred onto a pre-silanized coated slide. Silane coating was used as a surface treatment to improve the adhesion of sperm onto the slide. Sperm smears were incubated for 5 min with 200 µl of 10% formalin and washed for 5 min in 1 × PBS followed by 5 min in distilled water. To label midpiece and acrosome, slides were incubated for 15 min at room temperature in a humid chamber with 50 µl of MitoTracker (Green) dye (MitoTracker: 1:50 in 1 × PBS) and washed again with 1 × PBS and distilled water for 5 min each. MitoTracker Green labels mitochondrial proteins (and thus midpiece) and to a lesser degree also proteins in endoplasmic reticulum and Golgi microsomes or cytoplasm^87^, which can be utilised for acrosomal content labelling. Finally, the slides were mounted with the antifade medium Vectashield (Vector Laboratories) containing 4′,6-Diamidino-2-phenylindole (DAPI) for visualization of the nucleus.

Transmitted-light and fluorescence images of sperm cells were acquired on Leica TCS SP8/DM6 CFS upright confocal microscope using 63 × /N.A. 1.4 oil-immersion objective and lasers with wavelength 405 and 488 nm to excite DAPI and MitoTracker, respectively. A picture of the whole sperm and a more detailed picture of the sperm head were taken for each sperm. The selected sperm traits were measured with ImageJ software (ImageJ 1.50i^88^). The raw morphological measurements are provided in the Supplementary Table S6. To calculate the within-sperm repeatability of the measurements, ten sperm from two individuals of each species were independently measured three times.

#### Scanning electron microscopy

Sperm samples stored in paraformaldehyde were centrifuged (25,000 rpm, 7 min at 25 °C) to obtain the pellet of cells, which was gently re-suspended in a fixative solution containing 3% glutaraldehyde and 1% formaldehyde in 0.1 M cacodylate buffer (freshly mixed from stock solutions) and stored for 1 h on ice. After fixation, sperm were dripped onto a poly-l-lysine-coated coverslip (freshly coated high-precision coverslips) and dried at 40 °C. The coverslips with the sperm were washed three times for 5 min in 0.1 M cacodylate buffer and dehydrated by a graded series of ethanol treatments (30, 50, 70, 90, 96 and 100%). The slides were then rinsed with acetone and processed using the Critical Point Drying method (Leica EM CPD300) with acetone^89^. Finally, sperm smears on coverslips were placed on a sample stub using conductive carbon adhesive tape and coated with 7 nm of platinum using High Vacuum Coating System (Lecia EM ACE600). Sperm cells were imaged using an FEI Helios NanoLab 660 G3 UC. The secondary electrons were captured using Through-lens detector at 1 kV and 0.1 nA (Supplementary Fig. S4). The raw morphological measurements are provided in the Supplementary Table S6.

### Sperm swimming performance

In all experiments, sperm swimming performance was recorded at 100 × magnification using a microscope (C40, Olympus) with an installed camera (UI‐1540‐C, Olympus). The microscope had a preheated (40 ℃) thermal plate (Tokai-Hit, Japan) to avoid reduced sperm performance due to low temperature. Sperm swimming was recorded immediately after sample collection, and sperm were maintained at 40 °C throughout processing.

Firstly, we recorded the sperm swimming of both species in Advanced DMEM (Invitrogen). The collected ejaculate (0.5–1 µl) was diluted in 5 µl of DMEM preheated to 40 °C. Then 2.8 µl of the sample was transferred onto a standard 20 µm Leja count slide (Leja, The Netherlands), where the sperm were recorded for 15 s using three 4–6 s intervals at three different sites to record different sperm.

Secondly, we recorded CN and TN sperm in the PBS with and without the addition of the CN female fluid (Fig. 5). To perform the experiment, the freshly collected male ejaculate was diluted in 5 µl of PBS preheated to 40 ℃ to create a stock solution. Then, 2 µl of the sperm solution was transferred to both 5 µl CN female fluid and 5 µl PBS tube, thawed after being frozen in liquid nitrogen, and pre-warmed to 40 °C. Finally, the sperm solution was transferred to a Leja microscope slide with two chambers. Both chambers were alternately recorded, twice for 10–30 s each. The start times of recordings in each chamber were noted to control for their potential effects on sperm velocity. The order of the first chamber, i.e., fluid or PBS, was alternating between the experiments.

Sperm recordings were analysed using the computer-assisted sperm analysis (CASA) system, CEROS (Hamilton Thorne Inc., USA). Since there was no egg or other sperm attractant in the experiment, the sperm trajectory was not expected to be linear. Therefore, the average velocity of sperm in each sample was estimated using the curvilinear velocity (VCL), which is the average velocity of the sperm head through its path^90^ and is commonly used as a measure of in vitro sperm velocity in passerine birds^91^. To measure the VCL, the set image capture rate was 25 frames per sec and to maximize the data quality we only used cells with smoothed-path velocity > 5 μm s^−1^ or straight-line velocity > 10 μm s^−1^^76^. This allows to exclude static objects including non-moving sperm cells. In addition, the total number of the motile sperm (i.e., with smoothed-path velocity > 5 μm s^−1^ or straight-line velocity > 10 μm s^−1^) in each sample was calculated. The raw data of the VCL and the number of motile sperm for both experiments are provided in the Supplementary Table S6.

### Proteomic analysis of the female reproductive tract fluid

A total of seven female fluid samples collected from four CN females were used in this analysis (three females run in duplicates and averaged, and one as a singlet). The samples were defrosted and vortexed at room temperature. Then 10 μl of each sample was transferred to new tubes to which we added 20 μl of PBS. Samples were vortexed for 1 min before being isolated with ProteoSpin detergent-free columns (Norgen Biotek). Female fluid samples were then precipitated using ice-cold acetone (1:4) and centrifuged (14,000 rcf, 10 min at 4 °C), followed by resuspension of the dried pellets in the digestion buffer (1% SDC, 100 mM TEAB–pH = 8.5). The protein concentration of each lysate was measured with the BCA assay kit (Fisher Scientific, Waltham, MA, USA). Cysteines in 20 μg of proteins were reduced and diluted to a final concentration of 5 mM TCEP (60 °C for 60 min) and blocked with 10 mM MMTS (i.e., S-methyl methanethiosulfonate, 10 min at room temperature). Samples were cleaved with trypsin (1/50, trypsin/protein) at 37 °C overnight and peptides were desalted on a Michrom C18 column. Finally, eluted peptide cations were converted to gas-phase ions by electrospray ionization and analysed on a Thermo Orbitrap Fusion (Q-OT-qIT; Thermo Fisher, Waltham, MA, USA) using the same conditions and setup as Kuntova et al.^92^.

### Statistical analysis

#### Comparison of sperm morphology

The statistical analyses were conducted in R 3.0.3^93^. The estimates of the within-sperm measurement repeatability were calculated from an intercept-only linear mixed models (LMMs) without accounting for any explanatory variable and male identity as random effect using the *rptR* package^94^ with 95% confident intervals (95% CI) and 1000 bootstraps. Pearson’s correlation coefficients among sperm traits were calculated using the function *cor*^93^ and *corrplot*^95^.

We used linear mixed models (LMMs) using the *lme4* package^96^ to test for the differences in individual sperm traits between species. Individual sperm traits were used as a response variable and the species identity (CN vs. TN) as an explanatory variable. As we measured multiple sperm per individual, the male identity was set as a random effect. The region (sympatry vs. allopatry) and the interaction between species and region were included into the models as covariates. Model assumptions were checked through visual analysis of residual model plots^97^ and all graphs were made using the package *ggplot2*^98^. The Benjamini–Hochberg^99^ correction was applied on all p-values to control for the false discovery rate due to multiple testing.

#### Analysis of sperm velocity

To test for the effect of species on sperm velocity in a DMEM cell culture medium, we performed a linear regression model with the VCL as the response variable and the species identity (CN or TN) as an explanatory variable. As previous studies found the influence of the number of motile sperm on sperm velocity^34,100^, we included the log-transformed count of motile sperm as a covariate in the model. The mean number of motile sperm per individual was 185 (min: 21, max: 637). Linear regression model was then also used to test whether there is an association between the log-transformed count of motile sperm and the species identity.

To evaluate the impact of female fluid on the velocity of conspecific vs. heterospecific sperm, we performed an LMM with the VCL as a response variable. The male species (CN vs. TN), environment (PBS vs. female fluid) and their interaction were the explanatory variables. The starting time of the record in each chamber and the log-transformed count of motile sperm were added as covariates. The mean number of motile sperm per male individual in the experiment was 233 (min: 22, max: 1037). As each sperm sample was tested twice (once in female fluid and once in PBS) and each female fluid sample was used twice (once with conspecific and once with heterospecific sperm), the male and female identities were both entered as random effects.

#### Analysis of female fluid proteomes

To pre-process the obtained proteomic data we used MaxQuant 1.6.34 software^101^. The false discovery rate (FDR) was set to 1% for both proteins and peptides, with a minimum peptide length fixed to seven amino acids. To obtain protein IDs, we used the Andromeda engine for the MS/MS mapping against the zebra finch genome (*Taeniopygia guttata,* UP000007754, 2021). Quantifications were performed using a label-free algorithm^101^ with a combination of multiple unique (min. 2) and razor peptides and the whole matrix was LFQ-normalized. We removed all proteins which were not quantified or had a median value of zero from further analyses. The final dataset consisted of 14,756 high-quality spectral matches to a total of 975 proteins. From those 563 had at least two unique peptides. The spearman rank correlation between all four biological replicates were relatively high (averaged rho = 0.72, P < 2.20E-16, range 0.68–0.82, Supplementary Fig. S5A). The *mixtools* package^102^ was used to analyse the protein distribution (Supplementary Fig. S5B) and to perform unsupervised clustering to generate mixture models allowing each protein to be assigned to a group of either lowly expressed, moderately expressed or highly expressed proteins. Then, we performed gene ontology searches for significantly enriched GO terms, using the *topGO* package^103^ with the classic *topGO* algorithm and a Kolmogorov–Smirnov test with default parameters. *topGO* terms were searched within the latest chicken database (September 2021) —org.Gg.eg.db—and all proteins from our dataset were used as a background. We used 'clusterProfiler' and 'enrichplot' for hierarchical clustering and visualization of the enriched GO terms^104^.

## Supplementary Information

Supplementary Information.

## Data Availability

The mass spectrometry proteomics data have been deposited to the ProteomeXchange Consortium via the PRIDE^105^ partner repository with the dataset identifier PXD036878 and 10.6019/PXD036878.
