# Supplementary material for: Sperm morphology and performance in relation to postmating prezygotic isolation in two recently diverged passerine species
Source: Sci Rep. 2022 Dec 24;12:22275. doi: 10.1038/s41598-022-26101-5 (PMC9789955; doi:10.1038/s41598-022-26101-5)
Supplement: Supplementary file 1 — Supplementary Information. [file 41598_2022_26101_MOESM1_ESM.docx]

**Supplementary material**

**Sperm morphology and performance in relation to postmating prezygotic isolation in two recently diverged passerine species**

**Manon Poignet ^1^, Lucie Baránková ^1^, Jiří Reif ^2,3^, Pavel Stopka ^1^, Romana Stopková ^1^, Michaela Frolikova ^4^, Emily R. A. Cramer ^5^, Arild Johnsen ^5^, Pavel Kverek ^6^, Tomasz S. Osiejuk ^7^, Katerina Komrskova ^1,4^, Tomáš Albrecht ^1,8^*, and Radka Reifová ^1^***

^1^ Department of Zoology, Faculty of Science, Charles University, Prague, Czech Republic

^2^ Institute for Environmental Studies, Faculty of Science, Charles University, Prague, Czech Republic

^3^ Department of Zoology, Faculty of Science, Palacký University, Olomouc, Czech Republic

^4^ Laboratory of Reproductive Biology, Institute of Biotechnology of the Czech Academy of Sciences,
BIOCEV, Vestec, Czech Republic

^5^ Natural History Museum, University of Oslo, Oslo, Norway

^6^ Vilová 246, CZ-294 02 Kněžmost, Czech Republic

^7^ Department of Behavioural Ecology, Institute of Environmental Biology, Faculty of Biology Adam Mickiewicz University, Poznań, Poland

^8^ Institute of Vertebrate Biology, Czech Academy of Sciences, Brno, Czech Republic

***** Corresponding authors: Radka Reifová (radka.reifova@natur.cuni.cz) and Tomáš Albrecht (albrecht@ivb.cz)

**Figure S1.** Plots of Pearson correlation coefficients between sperm traits measured on photos from confocal microscope for common nightingale and thrush nightingale.

**Figure S2.** Plots of Pearson correlation coefficients between sperm head traits measured on photos from scanning electron microscope for common nightingale and thrush nightingale.

**Figure S3.** Velocity (VCL) of common nightingale (red) and thrush nightingale (blue) sperm in the fluid from the reproductive tract of common nightingale (CN) females and in PBS.

**Figure S4.** Examples of sperm head morphologies visualised using scanning electron microscopy.

**Figure S5.** Analysis of proteomes of common nightingale fluids from distal part of the female reproductive tract.

**Table S1.** Within-sperm measurement repeatability of individual sperm traits.

**Table S2**. Full linear mixed models testing for the effect of species and region on individual sperm traits.

**Table S3.** Measurements of sperm traits in two nightingale species.

**Table S4.** Results of linear mixed models testing for association between sperm morphological traits and species.


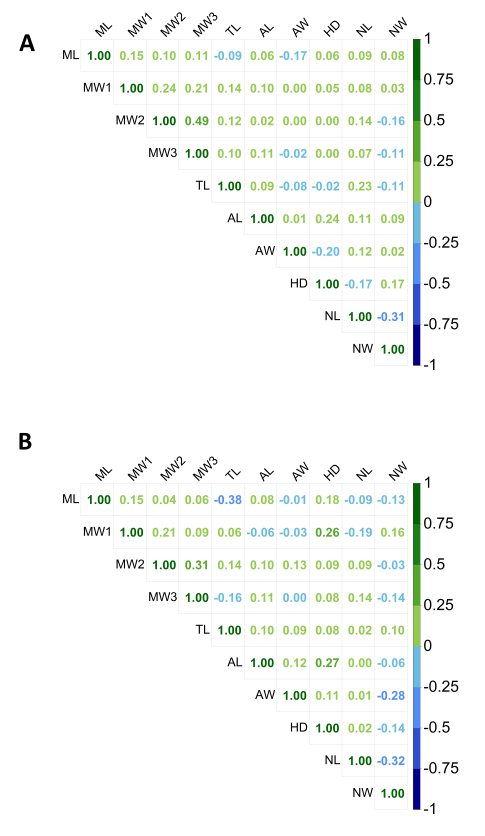


**Figure S1.** **Plots of Pearson correlation coefficients between sperm traits measured on photos from confocal microscope for common nightingale (A) and thrush nightingale (B).** The following measurements were correlated: midpiece length (ML), midpiece width (i) at the proximal tip (MW1), (ii) 100 µm from the proximal tip (MW2), (iii) at the distal tip (MW3), tail length (TL), acrosome length (AL), acrosome width (AW), helix distance (HD), nucleus length (NL) and nucleus width (NW). Correlation coefficients are displayed and associated with colour intensity (green gradient for positive values and blue gradient for negative values).


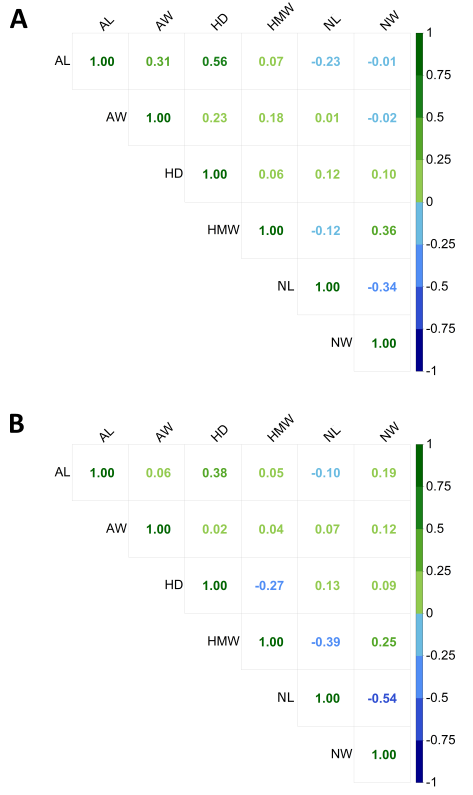


**Figure S2.** **Plots of Pearson correlation coefficients between sperm head traits measured on photos from scanning electron microscope for common nightingale (A) and thrush nightingale (B)**. The following measurements were correlated: acrosome length (AL), acrosome width (AW), helix distance (HD), helical membrane width (HMW), nucleus length (NL), and nucleus width (NW). Correlation coefficients are displayed and associated with colour intensity (green gradient for positive values and blue gradient for negative values).

**
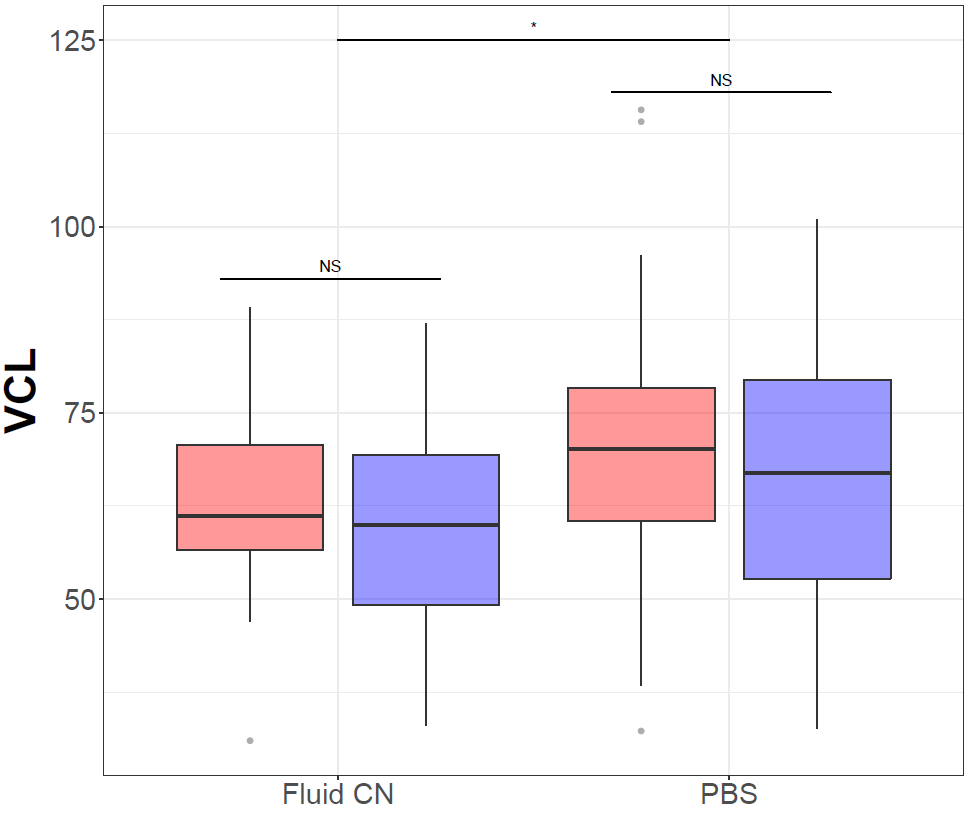
**

**Figure S3.** **Velocity (VCL) of common nightingale (red) and thrush nightingale (blue) sperm in the fluid from the reproductive tract of common nightingale (CN) females and in PBS.** Medians, quartiles, 1.5 quartile range, and outliers are represented. P-value: ns = P > 0.05; * = P ≤ 0.05; ** = P ≤ 0.01; *** = P ≤ 0.001.


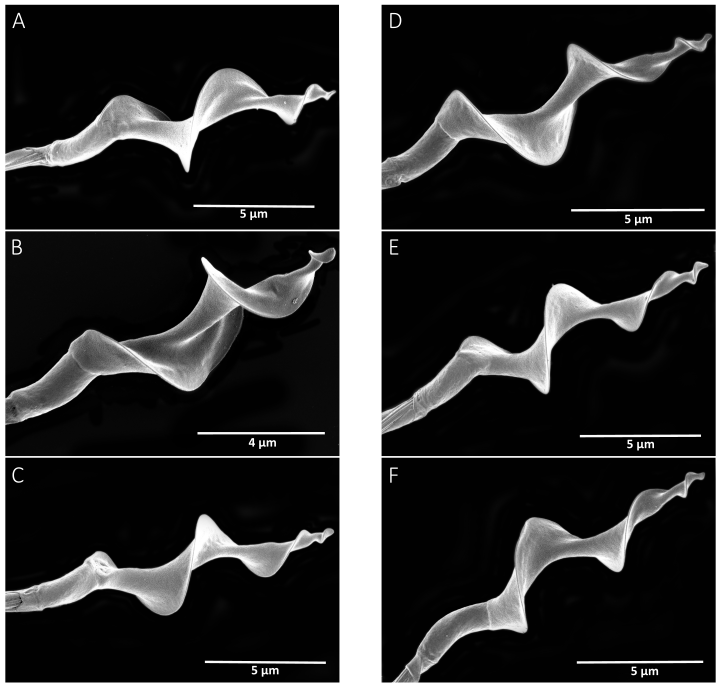


**Figure S4.** **Examples of sperm head morphologies visualised using scanning electron microscopy.** The images display sperm heads of (**A-C**) common nightingale and (**D-F**) thrush nightingale. The background of these images was modified to remove debris using Adobe Photoshop software, version CC 2017.

**
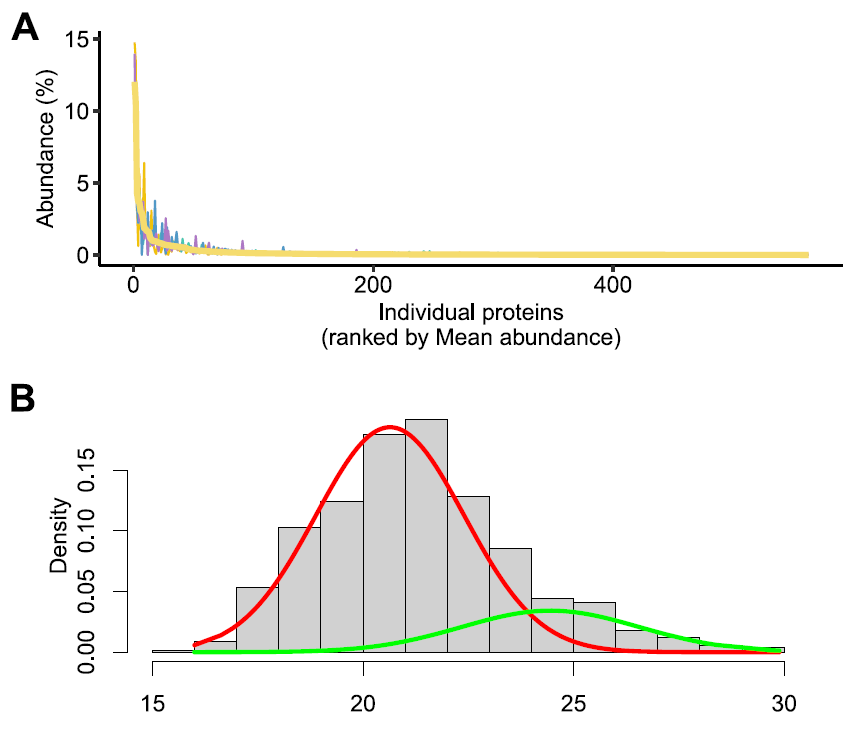
**

**Figure S5. Analysis of proteomes of common nightingale fluids from distal part of the female reproductive tract**. The protein rank-abundance plot for four biological replicates with the yellow line as the median value **(A)**. The mixture models (**B**) revealed that the data have n-binomial distribution with a mixture of at least two normal distributions.

**Table S1**. **Within-sperm measurement repeatability of individual sperm traits.** Repeatability was calculated based on three independent measurements of ten sperm in two individuals of each species (N=120 sperm) using linear mixed models without accounting for any explanatory variables (i.e., intercept-only model), with sperm identity as a random grouping variable. For each model, the repeatability, standard error (SE), and P value are shown.

|  |  | **Common nightingale** | | **Thrush nightingale** | |
| --- | --- | --- | --- | --- | --- |
| **Sperm traits** |  | **Repeatability ± SE** | **P** | **Repeatability ± SE** | **P** |
|  |  |  |  |  |  |
| Midpiece length |  | 0.92 ± 0.04 | < 0.001 | 0.92 ± 0.04 | <0.001 |
| Midpiece width 1 |  | 0.41 ± 0.14 | 0.002 | 0.36 ± 0.14 | 0.006 |
| Midpiece width 2 |  | 0.42 ± 0.14 | 0.002 | 0.40 ± 0.15 | 0.002 |
| Midpiece width 3 |  | 0.95 ± 0.02 | < 0.001 | 0.85 ± 0.06 | <0.001 |
| Tail length |  | 0.65 ± 0.14 | < 0.001 | 0.82 ± 0.07 | <0.001 |
| Acrosome length |  | 0.93 ± 0.03 | < 0.001 | 0.83 ± 0.07 | <0.001 |
| Acrosome width |  | 0.63 ± 0.12 | < 0.001 | 0.77 ± 0.09 | <0.001 |
| Helix distance |  | 0.30 ± 0.14 | 0.019 | 0.63 ± 0.12 | <0.001 |
| Nucleus length |  | 0.46 ± 0.13 | < 0.001 | 0.52 ± 0.13 | <0.001 |
| Nucleus width |  | 0.57 ± 0.12 | < 0.001 | 0.64 ± 0.12 | <0.001 |

**Table S2. Full linear mixed models testing for the effect of species and region on individual sperm traits.** The common nightingale and the allopatric region were used as the reference categories. Measurements were made on photos from confocal microscope. Estimates, standard errors (± SE), F-stats, P-values are shown for all models with significant P-values in bold.

|  |  | | |
| --- | --- | --- | --- |
| **Model terms** | **Estimate ± SE** | **F** | **P** |
| ***(i) Flagellum traits*** |  |  |  |
| **Midpiece length** |  |  |  |
| Intercept | 246.96 ± 1.72 | - | - |
| Species | -40.34 ± 2.43 | 523.7 | **<0.001** |
| Region | -1.37 ± 2.43 | 0.03 | 0.86 |
| Species x Region | 2.13 ± 3.43 | 0.38 | 0.54 |
| **Midpiece width** |  |  |  |
| Intercept | 0.97 ± 0.02 | - | - |
| Species | -0.01 ± 0.03 | 6.25 | **0.02** |
| Region | 0.04 ± 0.03 | 0.13 | 0.72 |
| Species x Region | -0.09 ± 0.05 | 3.71 | 0.06 |
| **Tail length** |  |  |  |
| Intercept | 18.42 ± 1.23 | - | - |
| Species | -1.79 ± 1.77 | 5.71 | **0.02** |
| Region | 1.07 ± 1.78 | 0.01 | 0.92 |
| Species x Region | -2.40 ± 2.50 | 0.92 | 0.34 |
| ***(ii) Head traits*** |  |  |  |
| **Acrosome length** |  |  |  |
| Intercept | 10.83 ± 0.16 | - | - |
| Species | 1.32 ± 0.23 | 63.25 | **<0.001** |
| Region | 0.11 ± 0.23 | 0.17 | 0.68 |
| Species x Region | -0.10 ± 0.32 | 0.08 | 0.78 |
| **Acrosome width** |  |  |  |
| Intercept | 4.36 ± 0.05 | - | - |
| Species | 0.23 ± 0.07 | 13.07 | **<0.001** |
| Region | 0.06 ± 0.07 | 0.03 | 0.85 |
| Species x Region | -0.10 ± 0.10 | 1.14 | 0.29 |
| **Helix distance** |  |  |  |
| Intercept | 5.45 ± 0.08 | - | - |
| Species | -0.15 ± 0.11 | 2.81 | 0.10 |
| Region | -0.04 ± 0.11 | 0.15 | 0.70 |
| Species x Region | 0.03 ± 0.16 | 0.03 | 0.88 |
| **Nucleus length** |  |  |  |
| Intercept | 2.81 ± 0.08 | - | - |
| Species | -0.01 ± 0.12 | 0.33 | 0.57 |
| Region | -0.04 ± 0.12 | 0.44 | 0.83 |
| Species x Region | 0.12 ± 0.17 | 0.51 | 0.48 |
| **Nucleus width** |  |  |  |
| Intercept | 1.38 ± 0.02 | - | - |
| Species | 0.03 ± 0.03 | 0.91 | 0.34 |
| Region | 0.02 ± 0.03 | 0.13 | 0.72 |
| Species x Region | -0.02 ± 0.05 | 0.17 | 0.68 |

**Table S3. Measurements of sperm traits in two nightingale species.** Measurements were made on sperms photos obtained by (**A**) confocal and (**B**) scanning electron microscopy. Means, standard deviations (sd), and minimum and maximum values are shown. Ten sperm per individual have been measured in 20 individuals per each species in case of confocal microscopy and five individuals per species in case of scanning electron microscopy.

**A**

| **Sperm traits**  **(confocal microscopy)** | | **Common nightingale** | | **Thrush nightingale** | |
| --- | --- | --- | --- | --- | --- |
|  |  | **Mean ± sd (µm)** | **Range (µm)** | **Mean ± sd (µm)** | **Range (µm)** |
|  |  |  |  |  |  |
| Midpiece length |  | 246.15 ± 8.34 | 203.87 - 266.17 | 206.93 ± 6.87 | 172.01 - 220.44 |
| Midpiece width 1 |  | 1.41 ± 0.15 | 1.01 - 1.87 | 1.40 ± 0.19 | 0.91 - 1.95 |
| Midpiece width 2 |  | 0.83 ± 0.16 | 0.51 - 1.24 | 0.74 ± 0.11 | 0.50 - 1.07 |
| Midpiece width 3 |  | 0.70 ± 0.17 | 0.16 - 1.22 | 0.64 ± 0.09 | 0.36 - 0.98 |
| Tail length |  | 19.08 ± 5.77 | 8.32 - 43.75 | 15.97 ± 4.95 | 6.19 - 39.64 |
| Acrosome length |  | 10.89 ± 0.79 | 8.90 - 13.23 | 12.16 ± 0.81 | 9.93 - 15.57 |
| Acrosome width |  | 4.39 ± 0.32 | 3.50 - 5.49 | 4.57 ± 0.28 | 3.50 - 5.36 |
| Helix distance |  | 5.43 ± 0.40 | 4.23 - 6.73 | 5.30 ± 0.46 | 4.12 - 6.94 |
| Nucleus length |  | 2.79 ± 0.38 | 1.84 - 4.23 | 2.84 ± 0.30 | 2.09 - 3.72 |
| Nucleus width |  | 1.39 ± 0.16 | 1.00 - 1.81 | 1.41 ± 0.16 | 1.00 - 1.85 |

**B**

| **Sperm traits**  **(scanning electron microscopy)** | | **Common nightingale** | | **Thrush nightingale** | |
| --- | --- | --- | --- | --- | --- |
|  |  | **Mean ± sd (µm)** | **Range (µm)** | **Mean ± sd (µm)** | **Range (µm)** |
| Acrosome length |  | 9.13 ± 0.51 | 7.87 - 10 | 9.81 ± 0.68 | 8.29 - 11.12 |
| Acrosome width |  | 3.52 ± 0.19 | 3.11 - 3.94 | 3.62 ± 0.16 | 3.24 - 3.91 |
| Helix distance |  | 4.38 ± 0.29 | 3.75 - 4.95 | 4.32 ± 0.32 | 3.64 - 4.99 |
| Helical membrane width |  | 0.94 ± 0.24 | 0.46 - 1.41 | 0.83 ± 0.21 | 0.38 - 1.27 |
| Nucleus length |  | 2.61 ± 0.33 | 2 - 3.2 | 2.41 ± 0.41 | 1.5 - 3.29 |
| Nucleus width |  | 1.15 ± 0.07 | 0.96 - 1.28 | 1.13 ± 0.07 | 0.95 - 1.3 |

**Table S4. Results of linear mixed models testing for association between sperm morphological traits and species.** The traits were measured on photos from scanning electron microscopy. The common nightingale was set as reference species. Estimate, standard error (SE), F-stats and P-values are shown for all models. Significant P-values in bold.

|  |  | | |
| --- | --- | --- | --- |
|  | **Estimate ± SE** | **F** | **P** |
| **Acrosome length** |  |  |  |
| Intercept | 9.13 ± 0.16 |  |  |
| Species | 0.68 ± 0.22 | 9.14 | **0.02** |
| **Acrosome width** |  |  |  |
| Intercept | 3.52 ± 0.06 |  |  |
| Species | 0.10 ± 0.08 | 1.76 | 0.22 |
| **Helix distance** |  |  |  |
| Intercept | 4.38 ± 0.08 |  |  |
| Species | -0.06 ± 0.11 | 0.29 | 0.61 |
| **Helical membrane width** |  |  |  |
| Intercept | 0.93 ± 0.05 |  |  |
| Species | -0.10 ± 0.06 | 2.52 | 0.15 |
| **Nucleus length** |  |  |  |
| Intercept | 2.61 ± 0.09 |  |  |
| Species | -0.20 ± 0.12 | 2.53 | 0.15 |
| **Nucleus width** |  |  |  |
| Intercept | 1.15 ± 0.02 |  |  |
| Species | -0.02 ± 0.02 | 0.70 | 0.43 |
